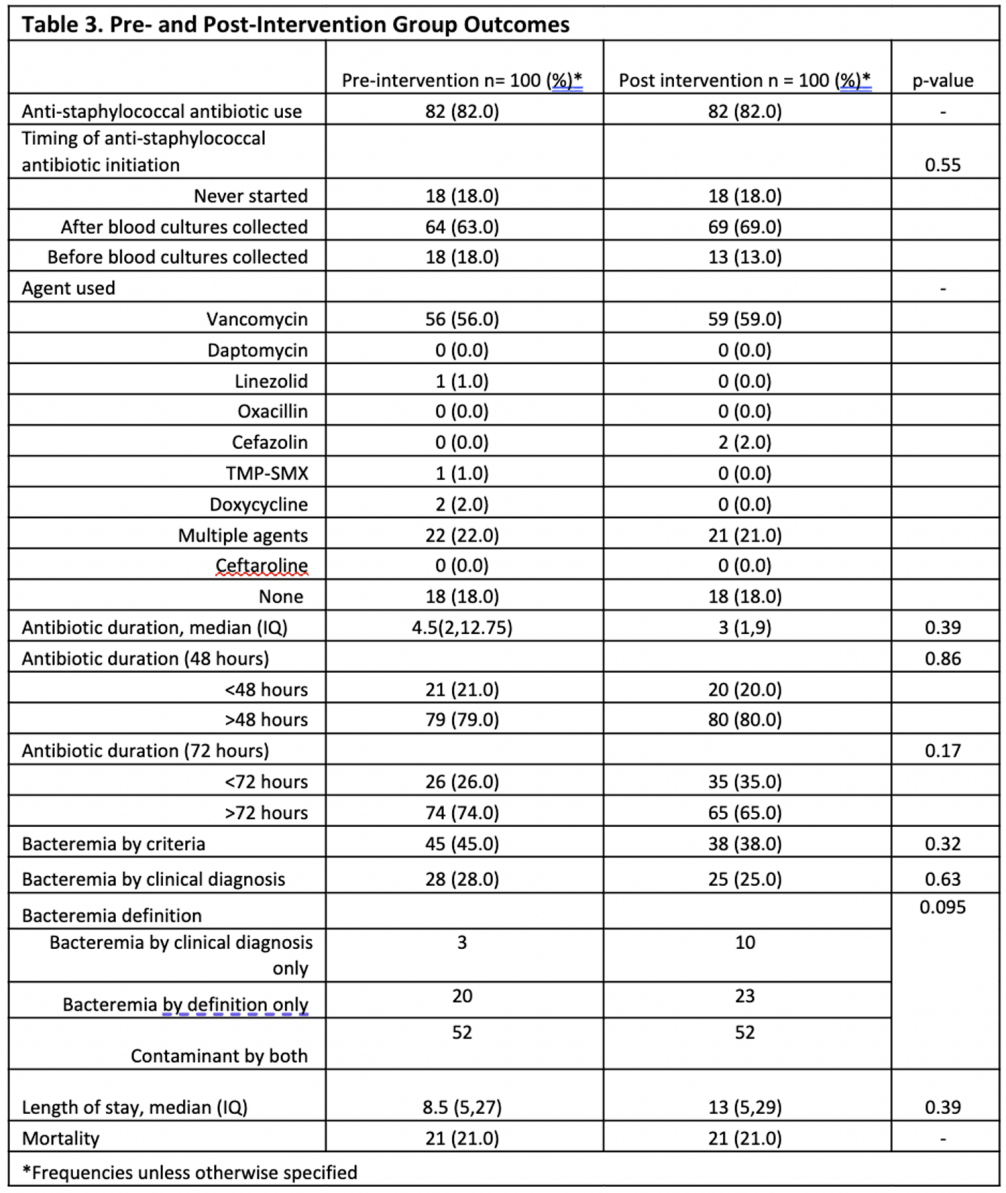# Evaluating the Clinical Impact of Species-Level Identification in Coagulase-Negative Staphylococci Positive Blood Cultures

**DOI:** 10.1017/ash.2024.162

**Published:** 2024-09-16

**Authors:** Ahmad Hamdan, Gabriela Andujar-Vazquez, Maureen Campion, Husain Poonawala, Catherine Cebulla, Majd Alsoubani

**Affiliations:** Tufts Medical Center

## Abstract

**Background:** Coagulase-negative staphylococci (CoNS) are often considered contaminants when isolated from blood cultures. While criteria exist to distinguish true bacteremia from contamination (Table 1), clinical judgement is often necessary. Clinical microbiology laboratories have traditionally identified CoNS to the species level only if present in multiple cultures. There have been concerns that blood cultures positive for rare or less familiar CoNS species might be misinterpreted as true bacteremia. Tufts Medical Center (TMC) clinical microbiology laboratory started reporting all CoNS to the species level in January 2023. We studied the impact of species-level identification of CoNS on clinically relevant outcomes following this change. **Methods:** The study evaluated inpatients at TMC aged ≥ 18 years with CoNS isolated from blood cultures between July 2022 and June 2023. The primary outcome was the difference in anti-staphylococcal antibiotic utilization between the pre- and post-intervention groups. Secondary outcomes included differences in true bacteremia diagnosis, length of hospital stay, and mortality between the two groups. We also compared the performance of Souvenir’s criteria with clinical judgement at distinguishing contamination from true bacteremia. A total of 100 patients were included in the pre- and post-intervention groups to detect an estimated effect size of 15% with a power of 81%. **Results:** Most patients were male, White, and English speaking (Table 2). No differences were found between the two groups in terms of infectious disease consultation frequency, blood culture collection department, or the presence of central venous catheters (Table 2). Staphylococcus epidermidis was the predominant CoNS in the post-intervention group. Blood cultures were repeated before and after starting antibiotics in 24% and 74% (pre-intervention) and 18% and 84% (post-intervention) of cases, respectively. Anti-staphylococcus antibiotic use was the same in both groups (82%). The median antibiotic therapy duration was 4.5 days pre- vs 3 days post-intervention (p =0.39). There were no differences in hospital length of stay or mortality between the two groups (Table 3). The clinical diagnosis of true bacteremia was established in 28% of cases in the pre- vs 25% in the post-intervention group (p= 0.63). Compared to clinical judgement, Souvenir’s true bacteremia criteria demonstrated a sensitivity of 80.3%, negative PV of 91.9%, and positive PV of 55.2%. **Conclusions:** Species-level identification of CoNS positive blood cultures did not impact antibiotic utilization, diagnosis of true bacteremia, length of hospital stay, or mortality. Further studies with larger cohorts and prospective designs are needed to validate these findings and assess the long-term implications in patients.

**Figure:**
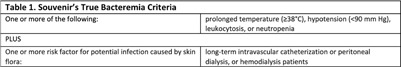

**Figure:**
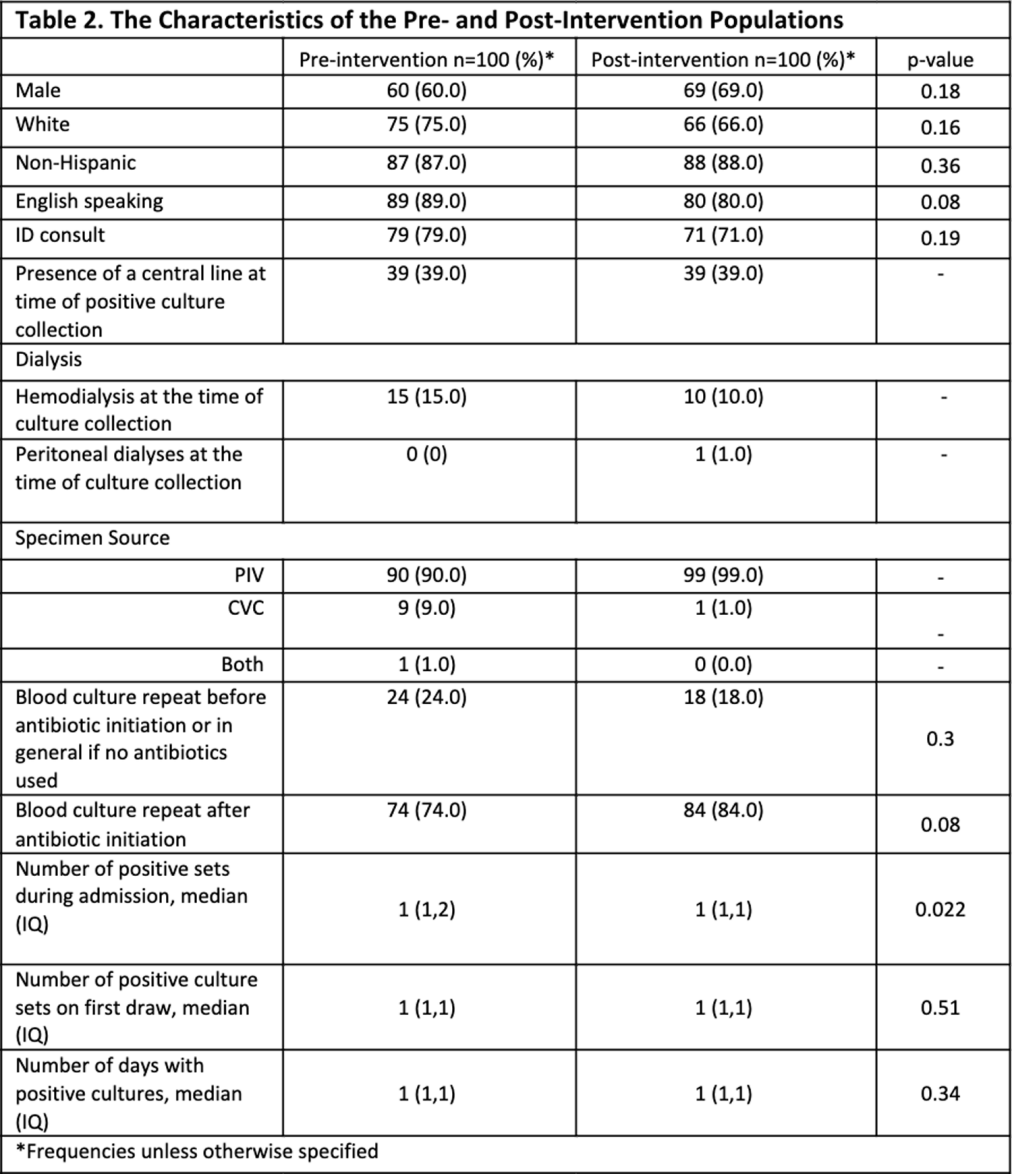

**Figure:**